# Understanding organisms by intuiting life: Kant, Goethe, and Steiner

**DOI:** 10.1007/s40656-025-00681-7

**Published:** 2025-07-16

**Authors:** Christoph J. Hueck

**Affiliations:** Akanthos Academy, Zur Uhlandshöhe 10, 70188 Stuttgart, Germany

**Keywords:** Organicism, Goethean science, Participatory science, Metamorphosis, Urpflanze, Johann Gottlieb Fichte, Intuitive understanding, Intellectual intuition

## Abstract

This paper investigates the enduring philosophical challenge of how a living organism may be understood, through the epistemological perspectives of Immanuel Kant, Johann Wolfgang von Goethe, and Rudolf Steiner. Kant’s analysis of the necessity of judging organisms as purposive and self-generating wholes is presented as foundational to any systematic account, insofar as it addresses the very conditions under which an organism can become an object of cognition. However, due to Kant’s strict separation of sensory intuition from conceptual understanding, he regarded purposive self-generation as merely heuristic, lacking causal legitimacy within empirical nature. In contrast, Goethe’s participatory and intuitive method, articulated in *The Metamorphosis of Plants*, integrates empirical observation with imaginative reproduction to achieve an intuitive grasp of an organism’s life and transformation. Conceived as a dynamic bridge between perception and concept, Goethe’s approach was subsequently interpreted and philosophically developed by Steiner. Steiner argued that an organism’s essential nature can be apprehended through a productive, intuitive mode of cognition that mentally reconstructs the organism’s formative principles and self-generative force. His position, which bears affinities to Fichte’s notion of intellectual intuition, both elucidates and extends Goethe’s method by asserting that the organism’s formative force is accessible through active, intuitive cognition. Thus, Steiner demonstrated how Goethe’s approach transcends Kant’s limitation on the knowability of organic life, enabling the empirical observation of spiritual efficacy in material nature. This paper ultimately contends that the Goethe-Steiner method offers an empirical yet intuitive framework and method for understanding the life and formation of living organisms.

## Introduction

Living organisms pose a profound challenge to both natural science and philosophy. They are physical, material entities, yet their intricate, self-organizing and developing structures appear as if shaped by purposeful, intelligent design. Given that the essential characteristics of organisms have thus far resisted materialistic and mechanistic explanation (Noble & Noble, 2023; Woese, 2004), one may either persist in the hope that future developments will yield such an explanation or concede that the problem of the organism lies beyond the reach of human cognition. Alternatively, one might posit the existence of non-material, holistic forces and laws that dynamically shape and animate organic matter. Such a hypothesis, however, could only be regarded as scientifically credible if it could be validated through supporting laws and empirical proof (Chen, 2024).

Although the question of the organism was believed to have been settled in the latter half of the twentieth century through the synthesis of Darwinism with statistical and molecular genetics (Mayr & Provine, 1980), the past two decades have seen growing criticism that reignited debate in both biology and philosophy, prompting a renewed consideration of alternative perspectives (for a comprehensive and up-to-date account, see Rosslenbroich, 2023). Scientists and philosophers focus once again on organismal properties like self-generation, purposiveness, autonomy, agency, and processuality (Meincke, 2019; Moreno & Mossio, 2015, p. 12; Švorcová, 2024), which were already discussed a century ago by vitalists like Hans Driesch (Chen, 2023) and Henri Bergson (Koutroufinis, 2023), or by so-called organicists like Edward S. Russell, Joseph H. Woodger, Ludwig von Bertalanffy, and others (Nicholson & Gawne, 2015). Some authors even refer to contributions that have been made by thinkers of German Idealism around the turn of the nineteenth century (Gambarotto & Mossio, 2022; Gambarotto & Nahas, 2023; Huneman, 2017; Michelini, 2008). In keeping with this renewed engagement with earlier insights, it has recently been proposed that Johann Wolfgang von Goethe’s conception of the organism as a continuously unfolding process may offer a significant contribution to the contemporary discourse (Rupik, 2024).

In this paper, I argue – by examining the philosophical interpretation of Goethe’s approach by the Austrian thinker Rudolf Steiner – that Goethe attained a direct, intuitive grasp of the formative laws and vital self-generating force of the organism. In Sect. 2 I start with an outline of Immanuel Kant’s analysis of the conditions and limitations of knowing an organism, as it provides a foundational basis for the following systematic inquiry. In Sect.  3, I describe Goethe’s treatment of the organism in his treatise *The Metamorphosis of Plants* (FA 24:109–152),^1^ with particular emphasis on its epistemological implications. In Sect. 4, I outline the key characteristics of Goethe’s phenomenological and intuitive approach, demonstrating how it bridges the divide between experience and idea. In Sect. 5, I offer a detailed exposition of Goethe’s intuitive mode of cognition as it pertains to the “primordial plant.” In Sect. 6, I discuss Steiner’s interpretation of Goethe’s approach in relation to Kant’s epistemological framework. I argue that Steiner offers a rigorous philosophical account that clarifies how Goethe’s method discloses the essential nature of the organism. In Sect. 7, I propose that Steiner’s reading of Goethe bears some resemblance to Johann Gottlieb Fichte’s concept of intellectual intuition, and thus enables an understanding not only of the organism’s formative laws but also of its self-generating force. Finally, in Sect. 8, I conclude by asserting that the Goethe–Steiner approach facilitates empirical cognition of the holistic laws and force that shape and animate living beings.

Goethe’s method and his conception of the *Urpflanze* have been described and discussed by numerous authors (cf. below, footnote 4). Here, I offer a systematic summary of this method which serves as a foundation and illustration for Steiner’s interpretation of Goethe’s method and way of “seeing” (*Anschauung*). Steiner placed particular emphasis on the *active* dimension of Goethe’s view of nature. According to Steiner, Goethe arrived at an intuitive perception of the formative force at work in the plant by re-enacting its developmental formation within dynamically changing mental images. I argue that this mode of perception is closely related to Johann Gottlieb Fichte’s concept of intellectual intuition, insofar as the intellectual activity that recreates the plant’s development intuits the ontologically operative formative force inherent in the plant itself.^2^

Although Goethe’s morphology must be seen within the broader context of the emergence of biology as an independent science at the turn of the nineteenth century, my concern here is not historical but systematic. The important contributions of figures such as Linné, Buffon, Blumenbach, Wolff, Kielmeyer, Herder, Schelling, and others have been extensively documented and discussed elsewhere (Reill, 2005; Richards, 2010; Steigerwald, 2019; Zammito, 2017).

## Kant’s epistemological analysis of the organism-problem

Kant’s analysis can be seen as foundational for any systematic approach to the problem of the organism, as he addressed the very conditions under which an organism can be apprehended and why its explanation is problematic at all. In the *Critique of Judgment*, Kant demonstrated that we can know an organism only by judging it as a purposive and integrated whole – just as we can make sense of a regular hexagon drawn in the sand only by attributing its origin to the concept-guided action of an intelligent agent (Kant, 2008, AA V:370). A bird’s wings, tail, and hollow bones, for instance, become intelligible only when understood in light of the purpose of flight, for otherwise they would appear entirely contingent (AA V:360). Thus, even the “intrinsic possibility” of an organism “emphatically presupposes the idea of a whole as that upon which the very nature and action of the parts depend” (AA V:408).

Yet while Kant acknowledged that we experience purposiveness – i.e., action guided by concepts – within ourselves (AA V:361), he insisted that we cannot ascribe this kind of causality to nature. For Kant, nature is “the sum of objects of the senses,” not an “intelligent being” (AA V:359), and therefore cannot be said to act according to concepts. Consequently, although we must judge organisms as purposive in order to understand them, we cannot attribute this purposiveness to nature itself. Rather, according to Kant, we project purposiveness onto our experience of natural objects, employing it as a regulative principle that enables us to comprehend organic form and function.

“[S]trictly speaking, we do not *observe* the ends [purposes] in nature as designed. We only *read* this concept *into* the facts as a guide to judgment in its reflection upon the products of nature. Hence these ends are not given to us by the object” (AA V:399).

Kant further contended that our discursive mode of thought is inherently incapable to comprehend how a natural whole can determine its parts. While we are able to understand how a whole might emerge from the interaction of its parts through what he called “a mechanical kind of generation” (AA V:408), we lack – according to Kant – the *intuitive* intellect necessary to apprehend the purposive and holistic organization of an organism directly and immediately (AA V:407).

Finally, Kant asserted that – since organisms are “nevertheless given in nature” (AA V:405) –, we are compelled to ground their purposiveness in “the supersensible substrate of nature,” a domain to which, however, “all possible insight is cut off from us” (AA V:410).

In summary, Kant demonstrated that organisms must necessarily be judged as purposive, self-generating wholes. However, he maintained that we cannot explain how such entities are possible in nature, since purposiveness and organic wholeness presuppose concepts or ideas – and therefore cannot be regarded as natural causes. For Kant, the very conditions that make organisms intelligible place them beyond the scope of mechanistic explanation, requiring us to invoke a principle that, while necessary for judgment, cannot be grounded in empirical (physical) nature itself.

Kant’s analysis continues to provoke discussion among philosophers of biology. Some interpret his account as offering a heuristic framework that ultimately serves to enable mechanistic explanations of organisms (Breitenbach, 2009; Ginsborg, 2001; Quarfood, 2006), upholding the view that only mechanistic accounts qualify as “proper science” (Van den Berg, 2014). Others argue that Kant’s work opens the door to recognizing purposiveness as a legitimate (Gambarotto & Nahas, 2022), and even necessary (Toepfer, 2012), concept within the natural sciences. Still others regard Kant’s position as an “unstable middle ground” between teleological and mechanistic approaches (Weber & Varela, 2002, p. 99). Yet as long as purposiveness and the self-organizing wholeness of living beings cannot be fully accounted for by scientific explanation, the organism remains, in Kant’s words, “a stranger in natural science” (AA V:390).^3^

## Goethe’s alternative approach to the organism-problem.

For Goethe, engaging with Kant’s *Critique of Judgment* marked “a most joyful period” of his life (Goethe, 1987, FA 24:444), as it inspired in him the notion of an intuitive understanding, a mode of inquiry he had genuinely developed through his natural scientific approach (FA 24:448).^4^ Goethe asserted that he had surpassed the epistemological constraints identified by Kant by cultivating a unique mode of sustained, attentive observation, coupled with what he called an “intuitive power of judgment” (*anschauende Urteilskraft*), particularly in relation to living beings (FA 24:447–448). To elucidate this concept, I will briefly outline Goethe’s morphological approach.

Goethe’s *Attempt to Explain the Metamorphosis of Plants* appeared at the same time as Kant’s *Critique of Judgment*.^5^ In this concise treatise, he articulated a general principle governing the formation of flowering plants – an insight he had arrived at during his Italian journey of 1786–87, which he referred to as the discovery of the “archetypal plant” (*Urpflanze*). Rather than describing and categorizing plants in terms of fixed properties (as was strongly promoted at that time by Linnaeus’ *System Naturae* [Larson, 1967]), Goethe conceived of it as an unfolding process, characterized by a dynamic rhythm of transformation. He identified three cycles of expansion and contraction: from seed to the leaf-bearing shoot, from calyx to corolla, and from carpels to fruit, returning ultimately to seeds again.^6^ Central to Goethe’s view was the search for unity amid morphological diversity. He maintained that the various parts of the plant are transformations of “the same organ,” which “fulfills nature’s laws throughout” (FA 24:149):

“The organ that expanded on the stem as a leaf, assuming a variety of forms, is the same organ that now contracts in the calyx, expands again in the petal, contracts in the reproductive apparatus, only to expand finally as the fruit” (FA 24:149).^7^

He referred to this unifying principle as “the leaf,” though he promptly acknowledged the limitations of this term, cautioning that “we would obviously need a general term to describe this organ that metamorphosed into such a variety of forms” (FA 24:150–151).

For Goethe, “the leaf” was not a physical structure, but a symbolic expression of a deeper, generative principle – one that manifested itself in diverse shapes throughout the plant’s development. This idea, which he also called a “transcendental main concept [*Hauptbegriff*]” (FA 24:91), combined with the concept of metamorphosis, allowed him to trace a continuous, formative logic underlying the plant’s apparent variability.^8^

*The Attempt to Explain the Metamorphosis of Plants* is a work of natural science rather than philosophy, yet Goethe’s search for the archetypal plant was, from the outset, also an epistemological endeavor.^9^ During his 1786 journey across the Alps to Italy, he observed that plants varied in form depending on their environmental conditions. These variations inspired him to look beyond the surface diversity and seek the underlying principle common to all plants:

“Since they all can be gathered under one concept, it became clear and clearer to me that the view [*Anschauung*] could be further enlivened in a higher way, a demand that I envisioned in the sensual form of a supersensible archetypal plant [*Urpflanze*]” (FA 24:748).

After a visit to the public garden in Palermo in April 1787, Goethe noted about the different plants:

“In the face of so many new and renewed forms, the old idea occurred to me again: whether I could not discover the archetypal plant among this multitude? After all, it must exist! *How could I otherwise know* that this or that form was a plant if all were not built according to the same model?” (Goethe, 1993, FA 15/1:286; emphasis added).

Similarly, he wrote in a letter to Charlotte von Stein in July 1786:

“It is no dream, no fantasy; it is an *becoming aware of the essential form* with which nature only ever plays, as it were, and playfully brings forth the manifold life” (Goethe, 2000, WA IV:7/2337).

Each of these quotes reveals Goethe’s acute awareness of his own cognitive processes and experiences – an awareness more commonly associated with philosophers than with natural scientists. Yet this underscores how Goethe consistently integrated external observation with internal experience in his entire mode of perception. His epistemological sensitivity is likewise apparent in his first conversation with Friedrich Schiller in 1794. With an implicit reference to Kant, he claimed that “nature” could be viewed as “effective and alive, striving from the whole into the parts” and sketched out his concept of the *Urpflanze*, claiming that it resulted from experience. Schiller as a trained Kantian objected that it was not an experience but an idea, but Goethe famously responded: “It can be very dear to me that I have ideas without knowing it, and even see them with eyes” (FA 24:437).^10^ Goethe thus claimed to have gained an *intuitive insight* into the generative principle of the plant with the “mind’s eye,” which Kant had declared impossible.

It is important to emphasize that Goethe was not looking for detached, Platonic ideas to explain nature. For him, seeing with the “mind’s eye” was not a retreat into abstraction, but a means of deepening one’s clarity about physical reality. In a note concerning Caspar Friedrich Wolff,^11^ he emphasized that “the eyes of the mind must work in constant, living union with the eyes of the body, because otherwise one runs the risk of seeing and yet seeing past” (FA 24:432). In fact, the metamorphic changes which he described in *The Metamorphosis of Plants* cannot be seen with physical eyes. The “eyes of the body” perceive discrete physical forms, yet their metamorphosis can only be apprehended through active imagination (Brady, 1984; Förster, 2002; Grave & Maatsch, 2014; Hueck, 2025). Goethe appears to have experienced these transformations within his imagination as vividly and concretely as he saw the plant’s shapes with his physical senses. And he was aware that both ways of seeing are required and must complement each other to understand the nature of organic life.

Goethe’s claim to intuit the archetypal plant as an objective reality through the “mind’s eye” clearly distinguishes his view from Kant, who would have understood the *Urpflanze* as an abstract and merely heuristic construct. Whereas Kant denied the possibility of intuitive knowledge of an organism (AA V:407), Goethe asserted that nature could be grasped as productive “from the whole into the parts.” His famous rejoinder to Schiller thus strikes at the core of Kant’s argument.^12^

The fundamental difference between Kant and Goethe lies in their understanding of the relation between ideas and nature. For Kant, the idea of the organic whole serves only a regulative function: it guides our investigation, but cannot be considered a genuine cause within nature, since ideas are not causally efficacious in the physical realm. Goethe, by contrast, developed a mode of inquiry in which perception and thinking were not held apart but united in a “living union.” This integration enabled him to experience the organic whole not as a conceptual fiction but as an objective reality (Förster, 2001; Nassar, 2015).

Moreover, whereas Kant analyzed the purposiveness and self-organization of organisms in abstract, philosophical terms, Goethe devoted himself to the meticulous study of real plants in their full, empirical richness. Reflecting on this lifelong engagement, he wrote that “by contemplating an ever-creative nature,” he had made himself “worthy of mental participation in her productions” (FA 24:447–448). In claiming this participatory knowledge, Goethe asserted that he had overcome the epistemic divide between sense perception and concept, between experience and idea – a divide which, for Kant, rendered the natural causality of organisms ultimately unknowable.^13^

## Goethe’s natural scientific method

Can it be said that Goethe actually developed a viable, transparent, communicable, and reproducible method for understanding the organic? And what, precisely, does this method entail? While Goethe’s scientific approach has frequently been addressed in general terms (for recent historical overviews see Richards, 2010 and Zammito, 2017; for an extended discussion cf. Breidbach, 2006; Rupik, 2024; see also Amrine, et al., 1987; Bortoft, 1996; Bortoft, 2012; Seamon & Zajonc, 1998, and further references in footnote 4), only few scholars have treated it as a practical and trainable method (cf. Brook, 1998; Hueck, 2023b; Irwin & Baxter, 2008; Wahl, 2005). In what follows, I offer a summarizing account of Goethe’s approach as a systematic sequence of four interrelated stages, through which one may progress from empirical observation to the intuitive apprehension of the idea. This exposition of Goethe’s method will provide the foundation for Steiner’s philosophical interpretation, which will be discussed in Sects. 6 and 7.

Goethe’s method begins with careful, sustained, and committed observation, coupled with the thoughtful collection and study of the phenomena under investigation.^14^ This foundational stage emphasizes *direct sensory engagement* with nature, unprejudiced by theoretical assumptions, and aims to let the phenomena reveal themselves in their own terms.

Goethe’s method then advances to *imaginative transformation* of phenomena through what he termed “exact sensorial imagination” (*exakte sinnliche Phantasie*) (Bortoft, 1996; Simms, 2014; Vries, 2011). This process is particularly relevant in morphology, where the researcher traces and reproduces the metamorphic sequence that the organism displays. In doing so, one does not merely observe change, but participates in it by imaginatively living through the form’s development. Goethe described this with remarkable subtlety and introspective nuance:

“When I see a formed thing (*eine entstandene Sache*) and ask how it came into being and measure the path back as far as I can follow it, I become aware of a series of stages that I cannot see next to each other but must visualize in memory to form a certain ideal whole. At first I am inclined to imagine certain steps; but because nature does not make a leap, I am ultimately compelled to view the sequence of uninterrupted activity as a whole, by suspending the individual without destroying the impression” (FA 24:352–353).

Accordingly, to genuinely comprehend natural transformation, the scientist must cultivate a corresponding flexibility in thought and perception. As Goethe put it: “What is formed is immediately reformed, and if we want to achieve a certain degree of living observation of nature, we have to keep ourselves as mobile and flexible as the example with which nature proceeds” (FA 24:392; cf. Amrine, 1998).

Goethe’s method further leads to experiences that open the way to *insight into the formative laws* underlying natural phenomena. If, for example, one imaginatively reproduces the unfolding of a plant, one performs within the mind the same formative movements that the plant undergoes in nature. In doing so, the governing law of transformation reveals itself directly within the experience.^15^ As such, the imaginative reenactment of nature’s processes becomes the central methodological means of understanding them (cf. Förster, 2012, pp. 250–276). However, because this is the point at which perception begins to give way to judgment, Goethe issued a caution:

“[O]ne cannot be careful enough not to jump to conclusions too quickly: for it is in the transition from experience to judgment (…) that all of man’s inner enemies lie in wait for him, as it were at a pass: imagination, impatience, rashness, complacency, stiffness, thought-form, preconceived opinion, laziness, carelessness, changeability and whatever else the whole crowd may be called, all lie in ambush here and overwhelm both the acting man of the world and the silent observer, seemingly secure from all passions” (FA 25:30).

Finally, Goethe’s method facilitates to *intuit the essential principle* of an organism, e.g., the “leaf” in plant morphology:

“It had dawned on me that in that organ of the plant which we usually refer to as the leaf, the true Proteus lies hidden, able to hide and reveal itself in all formations. Forwards and backwards, the plant is always only leaf” (FA 15/1:346).

Goethe’s method thus rests on four key elements: (1) thorough sensory observation of distinct yet related natural phenomena – for example, stem-leaves, sepals, petals, and carpels; (2) their active, imaginative transformation into one another; (3) the experiential discernment of the laws governing these transformations, namely a threefold process of expansion and contraction; and (4) an intuitive apprehension of the essential principle that unifies the outwardly diverse phenomena – the so-called “leaf.”

More broadly speaking, Goethe’s method is characterized by receptivity, productivity, experiential engagement, and intuition. It culminates in the ability to apprehend natural phenomena from the whole to the parts through what he termed an “intuitive power of judgment” (FA 24:448).^16^

Goethe’s natural scientific approach bridges the rigid subject-object dichotomy characteristic of conventional scientific inquiry (Bauer, 2023; Pfau, 2010) and can therefore be described as *participatory*. In intuiting the essence of a phenomenon, the scientist no longer experiences himself as separated from the object of study. Goethe referred to this dimension as “rational amalgamating” (*rationelles Amalgamieren*):

“This would therefore, according to my experience, be the point where the human mind can approach objects in their generality most closely, bring them to itself, amalgamate with them (as we usually do in common empiricism) in a rational way, as it were” (FA 25:125–126).

In a similar remark, he once called this method “delicate empiricism” (*zarte Empirie*):

“There is a delicate empiricism that makes itself intimately identical with the object and thereby becomes the actual theory. But this enhancement of the intellectual capacity belongs to a highly educated age” (FA 25:113).^17^

In summary, Goethe sought to “carefully, patiently, and gingerly follow nature where it leads” (Rupik, 2024, p. 40), aiming “to comprehend the logic of nature *from within* instead of merely trying to represent or mimic it” (Petteni, 2022, p. 43). His method offers the possibility of attaining an empirically grounded, intuitive knowledge of the formative principle that underlies the self-organization of living beings.

## Cognizing the archetypal plant

Intuitive understanding in the Goethean sense – seeing an organism with the “mind’s eye” – focuses on the essential nature of an organism that lies beyond sensory perception. Once this essence is consciously apprehended, it cannot only be recognized in any organism, but may also serve as a basis for mentally envisioning entirely new ones. Accordingly, Goethe wrote of the *Urpflanze*:

“With this model and the key to it, one can invent plants ad infinitum that must be consistent, that is to say, they may not actually exist, but they could exist and (…) have an inner truth and necessity” (FA 15/1:346).

This compelling notion mentions two essential aspects of the archetypal plant: the “model” and the “key to it.” Förster proposed that the “model” may be understood as the *constructive element* of the *Urpflanze* – the “leaf” – while the “key to it” can be conceived as the *constructive rule*, namely the three cycles of expansion and contraction with progression (Förster, 2012, p. 274).

To further understand the concept of the “leaf,” it is helpful to consider the reflections of the Goethean biologist Andreas Suchantke, who described it as the principle of a living surface:

“In contrast (…) to animals, the surface of the plant is not a boundary that insulates its interior from the environment (…), but rather a transparent filter and zone of passage, where a dynamic exchange of substances occurs – substances that originate, on the one hand, from the surrounding atmosphere, (…), and on the other, from the (…) soil. Both (…) are infused with the life forces of the organic realm within this boundary layer and elevated to a higher state” (Suchantke, 1983, p. 377).

This description, which is closely aligned with biological facts, may serve as a foundation for contemplating Goethe’s *Urpflanze* with the mind’s eye. Consider, for example, the development of a flowering plant. Imagine the idea of a living surface, interacting with its elementary surroundings. Add the idea of development beginning with the division of a “living point,” an “ideal primordial body [*Urkörper*]”, which is “already divided within itself into two, for without the (…) division of the one, no third emergence can be conceived” (FA 24:354). Imagine this *Urkörper* (seed) diverging into the polarity of a supporting pole in the moist and dark (root) and a developing pole in the dry and light (green plant). Imagine a dynamic vertical extension (the plant’s growth between the earth’s gravity and the sun’s light) accompanied by a spiral movement of its lateral appendages (FA 24:786–790). Enliven this imagination with the dynamic idea of three cycles of expansion and contraction, the first progressing successively from seed to cotyledons to foliage leaves; the second unfolding adjacently from calyx to corolla to reproductive organs; and the third organized in a nested fashion, from carpel to fruit to seed (cf. footnote 6). Finally, integrate the modifying influence of environmental conditions on the plant’s formation (e.g., expanding in spring and summer, contracting in fall and winter).^18^

Taken together, this intricate interplay of dynamic, mutually implicating ideas constitutes the *living morphological principle* of the dicotyledonous plant.^19^ By “bringing it forth” in our mind, dynamically moving through it “forwards and backwards,” “we may say that we intuit [the plant] in a proper and higher sense, that it belongs to us, that we have attained a certain mastery over it” (FA 25:142). In other words, we have come to “see it with the mind’s eye.”

## Steiner’s explanation of Goethe’s morphological approach

The most extensive and philosophically rigorous engagement with Goethe’s natural scientific method, to the best of my knowledge, was undertaken by the Austrian philosopher Rudolf Steiner (1861–1925). Steiner, who is primarily known for his Anthroposophy, was not only an esotericist, spiritual researcher, teacher, and reformer, but also a philosopher and one of the earliest editors of Goethe’s scientific writings. In 1882, while studying natural sciences at the Technische Hochschule in Vienna, Steiner was recommended by the literary scholar Karl Julius Schröer to serve as editor of Goethe’s scientific works for the monumental *Deutsche National-Litteratur* series, edited by Joseph Kürschner. Steiner embraced this task with enthusiasm and scholarly rigor, publishing the first volume of Goethe’s *Natural Scientific Writings* with an extensive introduction in 1884. This was followed by three further volumes each introduced by Steiner (collectively published in Steiner, 1987, GA 001, an English translation of which is also cited here [Steiner, 2010, CW 1]). During that time, in which he also contributed to the *Weimar Sophien-Edition* of Goethe’s collected works, Steiner began publishing his own philosophical reflections on Goethe’s scientific method. His monographs *Outlines of an Epistemology of Goethe’s Worldview* (Steiner, 1979, GA 002), *Goethe’s Worldview* (Steiner, 1990, GA 006; Steiner, 1994, CW 6), along with several essays on Goethe’s approach to nature (Steiner, 1989, GA 030:227–232; GA 030:482–487; GA 030:265–288; GA 030:320–327; GA 030:69–85), represent a sustained and original attempt to articulate the epistemological foundations of Goethe’s science. Steiner also revisited this theme in his autobiography (Steiner, 1982, GA 028:112–117) and discussed it in many of his lectures.^20^

Steiner’s editorial work was met with praise for its originality, but also with some criticism for lack of editorial precision (Harnack, 1890; Mandelkow, 1980; Raub, 1965; Ziegler, 2018). Heinz Kindermann, reviewing more than 120 years of Goethe-reception, wrote that.

Rudolf Steiner’s (…) exploration of Goethe in these early works, not yet anthroposophically colored, is one of the most important pioneering works of Goethe research (…), despite all its one-sidedness. (Kindermann, 1966, p. 68, my transl.)

However, Steiner’s comments on Goethe were also met with skepticism and, at times, outright prejudice. For example, Astrida Tantillo wrote:

While many of Goethe’s earlier readers attempted to find religious overtones within the natural writings of their hero, the propensity of treating Goethe’s scientific texts as mystical rather than philosophical is (…) illustrated in the works by Rudolf Steiner (…) and his followers, the anthroposophists, (…) who often write on Goethe’s science, [and] tend to look within his texts for messages of personal/spiritual guidance and fulfillments. (Tantillo, 2002, p. x)

If such criticism were justified, it would undoubtedly hinder a fair and rigorous engagement with Steiner’s commentary on Goethe’s philosophy and science. However, there is nothing mystical in Steiner’s work on Goethe. His research was clearly intended to demonstrate how Goethe offered a meaningful response to the problems of knowledge that Kant had so sharply defined.^21^

Detailed academic studies of Steiner’s work on Goethe remain surprisingly scarce and have largely come from anthroposophically oriented philosophers (Schieren, 1998; Sijmons, 2008), physicians, and scientists (Kiene, 1984; Kranich, 2007; Penter, 1998; Sachtleben, 1988), with only a few exceptions among non-anthroposophical academics (Ginges, 2012; Goy, 2019; Raub, 1963; Smook, 1992; Vries, 2011; Ziguras, 2010, 2014). In what follows, I offer a discussion of Steiner’s interpretation of Goethe’s approach to the question of the organism. The aim is to clarify how Steiner understood the epistemological structure of Goethe’s method, and how he believed it ultimately overcame the conceptual challenges posed by Kant’s philosophy of biology.

In the introduction to the first volume of *Goethe’s Natural Scientific Writings*, Steiner claimed that Goethe had discovered “the nature of the organism” and that he had set forth

“the principle of how an organism manifests as it does, the causes leading to the outer expressions of life. Indeed, he illuminated everything related to the principles involved in such matters” (CW 1:1; GA 001:9-10).

The key aspect of Goethe’s approach, according to Steiner, is epistemological:

“What is significant in the metamorphosis of plants (…) is not the discovery of the single fact that leaf, calyx, corolla, and so on, are identical organs; rather, it is *the magnificent thought structure of a living whole consisting of mutually interpenetrating, formative principles*. This dynamic thought structure, which arises from that discovery, determines out of itself the details and individual stages of plant development. The greatness of this idea (…) dawns on us only *when we try to bring it to life in our own mind and attempt to rethink it*. That is when we become aware of how this thought *is the very nature of the plant itself, translated into the form of an idea, and living in our mind just as it lives in the object*” (CW 1:3: GA 001:12-13; emphasis added).

The interdependent, formative laws of the plant (such as the threefold rhythm of expansion and contraction with progression, the spiral and vertical tendencies, and others described in Sect. 4), which form a “living whole” (as described in Sect. 5), enable an understanding of the plant from the whole toward its parts, while the imaginative activity of the knower enlivens this lawful structure, “bring[s] it to life in one’s mind,” and thus – according to Steiner – opens a path to insight into the organism’s formative laws and force.

Steiner explained that the relationship between perceptions and concepts differs between the inorganic and organic realms. In the inorganic realm, sensual phenomena become intelligible through their connection with concepts:

“Knowledge of inorganic nature is based on the possibility of comprehending the external world through the senses and expressing its interactions through concepts. Kant regarded the possibility of knowing things in *this* way as the only kind of knowledge accessible to the human being. He called this kind of thinking discursive. *What* we wish to know is an external perception; the concept, or combining unity, is merely a means” (CW 1:50; GA 001:82).^22^

In the organic realm, however, phenomena cannot be connected in this manner. The seed cannot be regarded as the cause of the flower. The concept that links seed and flower must not be abstract, as in the inorganic domain – “borrowing its meaning” (CW 1:50; GA 001:82) from perceptible phenomena – but rather must be a concrete and generative entity in its own right, from which seed and flower alike – as well as all other organs and stages of development – can be derived:

“[A]ll sensory qualities arise in an organism as the result of something *no longer perceptible to the senses.* They appear as the result of a higher unity hovering over the sense-perceptible processes. It is not the form of the root that determines that of the stem, and not the form of the stem that determines that of the leaves, and so on. All of these forms are determined instead by something that exists above them and whose form is inaccessible to the senses. The perceptible elements exist for one another, but not as a result of one another. They are not mutually determined by one another but by something else. Here what we perceive with our senses cannot be reduced to other sensorial factors; we must include in our concept of events elements that do not belong to the world of the senses; *we must go beyond the sensory world.* What we *perceive* is no longer enough; to comprehend the phenomena, we must conceptually grasp the *unifying principle*” (CW 1:44; GA 001:73-74).^23^,^24^

These notions, once again, bear a striking resemblance to Kant. Just as Kant asserted that an organism’s “intrinsic possibility emphatically presupposes the idea of a whole as that upon which the very nature and action of the parts depend” (AA V:408), Steiner likewise emphasizes that the unity of the organism cannot be derived from sensory observation alone, but must be grasped conceptually.

Therefore, if

“we wish to comprehend organic nature, (…) we cannot apprehend the ideal, conceptual aspect as something that borrows its meaning by expressing or indicating something else; rather, we would have to apprehend the *ideal element as such.* It would have to contain its own meaning, stemming from itself, not from the spatial-temporal world of the senses. The unity that our mind merely abstracts in the case of the inorganic would have to build upon itself, forming itself *out of itself;* it would have to be fashioned according to its own being, not according to the influences of other objects” (CW 1:50; GA 001:82)

Here, Steiner explicitly moves beyond Kant. For Kant, concepts can derive their content *only* from sensory perception; insofar as they are given a priori (such as the categories or the concept of purposiveness [AA V:181]), they remain mere empty *forms* that enable experience. Steiner, by contrast, argued that this Kantian view applies only to concepts pertaining to the inorganic realm. In the cognition of the organic, he maintained, a different mode of thinking is required:

“What is needed to attain such comprehension? We need a kind of thinking that can give a thought a substance [*Stoff*] not derived from outer sensory perception, a thinking that comprehends not only what is perceived externally by the senses, but also apprehends pure ideas apart from the sensory world. A concept that is not abstracted from the sensory world but whose content [*Gehalt*] develops out of itself and only out of itself can be called an *intuitive concept,* and the comprehension of such a concept may be called ‘intuitive knowledge.’ What follows from this is clear: *A living organism can be comprehended only through an intuitive concept*” (CW 1:50; GA 001:82-83).^25^

Thus, our thinking must itself become productive if we are to grasp the generative principle of the organism. This idea arguably represents an original and significant contribution to the interpretation of Goethe’s morphology and to the broader inquiry into the nature of the organism.

In *Outlines of an Epistemology of Goethe’s Worldview* (Steiner, 1979, GA 002) Steiner described in more detail what he meant by intuitive cognition:

“Our mind must (…) work much more intensively in grasping the [organic] type than in grasping the [inorganic] natural law. It must generate not only the form, but also the content. It must undertake an activity that, in inorganic natural science, is performed by the senses and which we call intuition. At this higher level, the mind itself must therefore be intuitive. Our power of judgment must *view in thinking* and *think in viewing*. Here, as Goethe first set out, we are dealing with an intuitive power of judgment” (GA 002:109).

The crucial point here is that intuitive thinking is an active and generative faculty of mind, not a passive mode of perception. What the mind brings forth in intuition is the organism’s productive idea itself – the type or *Urpflanze* “living in our mind just as it lives in the object” (CW 1:3; GA 001:12).^26^

“This idea, which corresponds purely to the organic aspect of the organism, is the archetypal organism; it is Goethe’s *type.* Thus, the eminent validity of the idea of the type becomes apparent. It is not merely an *intellectual concept* but the truly organic aspect of every organism, without which it would not be an organism. Because it manifests in *every* organism it is more real than any actual, particular organism. It also expresses the essence of an organism more *fully* and *purely* than *any individual organism in particular.* The way we arrive at the idea of the type is fundamentally different from the way we arrive at a concept of an inorganic process, which is abstracted from external reality and is not active within it. The idea of the organism, on the other hand, works actively within the organism as its entelechy – it is the essence of the entelechy itself in a form apprehended by our reason. The idea is not a summary of experience; it *brings about* that which is to be experienced [*bewirkt* das zu Erfahrende]” (CW 1:51; GA 001:84-85).

The last sentence encapsulates Steiner’s core assertion, through which he surpasses other interpretations of Goethe and indeed even inaugurates a new methodological chapter in the study of nature. For how can something that exists solely “within our mind” (an idea) truly exert efficacy in nature and “bring about that which is to be experienced?” Here, Steiner argues for the possibility of recognizing spiritual efficacy within the material world – a stance that would not only resolve Kant’s problem of teleology, but also lay the foundation for an entirely new understanding of nature and of the human being’s relationship to it. In fact, Steiner goes so far as to assert the primacy of the spiritual over the material, claiming that the entelechical principle “is more real than any actual, particular organism.”^27^

Thus, in Steiner’s interpretation, it is not the sensually perceptible that constitutes the true and effective reality of the organism, as assumed in natural science, but rather its entelechial idea.^28^ When we actively apprehend this idea, we do more than model the organism’s formative principle – we mentally enter into its formative core, as it were, from which the organism *brings forth* its outward, perceptible form. It is not the sensory manifestations of the organism – its root, stem, leaves, flower, and so on – that reveal its essential principle; rather, this principle is apprehended in thinking as their unifying and productive idea, the “leaf.” And because the organism actively alters its appearance from within – undergoes development – the mental grasp of its organic principle must likewise be dynamic and inherently generative. Goethe alluded to this in claiming about the “archetypal plant:”

“With this model and the key to it, one can invent plants ad infinitum that must be consistent, that is to say, (…) they *could exist* and (…) have an inner truth and necessity” (FA 15/1:346; emphasis added).

Steiner contended that the causes of an organism’s structure and development cannot be found within the realm of the sensually perceptible. This view again aligns with Kant, who stated that “we do not observe purposes in nature;” they “are not given to us by the object” (AA V:399). Kant concluded that the principle of organismal purposiveness may reside “in the supersensible substrate of nature, all possible insight into which is, however, cut off from us” (AA V:410). Steiner, too, located the principle and cause of the organism in the supersensible realm, but in sharp contrast to Kant, he asserted that this realm is accessible – through the active mental (re)production of the organism’s formative processes.

Kant distinguished two elements of cognition: (sensory) intuitions and concepts. For Kant, intuitions can only be passively received, whereas concepts are actively (spontaneously) applied:

“Our cognition arises from two fundamental sources in the mind, the first of which is the reception of representations (the receptivity of impressions), the second the faculty for cognizing an object by means of these representations (spontaneity of concepts); through the former an object is given to us, through the latter it is thought in relation to that representation (as a mere determination of the mind)” (Kant, 1998, AA III:A 50).

From the overall context presented here, it follows that, on Kant’s view, it is not possible to grasp the essential nature of the organic. According to Steiner’s interpretation of Goethe, however, the essence of the organism becomes knowable when the passively received intuitions are permeated by an inner, spontaneous activity and are, as it were, recreated (“brought to life”) within the human mind. Conversely, in Goethe’s way of seeing, cognition does not judge by means of pre-established categories and concepts, but instead receives its (dynamic) conceptual forms from the organic phenomena.

In his autobiography, Steiner reflected on the explanatory power of Goethe’s approach by contrasting it with explanation in mechanics. He argued that

“mechanics satisfies the desire for understanding because it generates concepts in the human mind in a rational way, which it then finds realized in the sensory experience of the inanimate. Goethe [was] the founder of an organic science that relates to the animate in the same way. (…) If one lets [the forms of a plant] emerge from each other in one’s mind, one constructs the whole plant. One recreates in one’s mind the process by which nature actually creates the plant” (Steiner, 1982, GA 028:113-114).

Through this act of re-creation, one consciously participates, as it were, in the genesis of the plant. This is what Goethe meant when he wrote that he had made himself “worthy of mental participation in her productions by contemplating an ever-creative nature” (FA 24:448). And because one participates mentally in the plant’s coming-into-being and experiences the laws governing its formation, one comes to understand how the plant has arisen.

By drawing attention to this “rational construction” of the *Urpflanze*, the results of which can subsequently be rediscovered in the perception of physical plants, Steiner made it clear why Goethe’s way of cognition explains the *Urpflanze* in Kant’s sense. For Kant had stated that explanation is “derivation from a principle which must (…) be capable of being clearly cognized and specified” (AA V:412), because “we have complete insight only into what we can make and accomplish according to our concepts” (AA V:384).

The Goethean idea of the organism is an active, mental reconstruction of the principles underlying the organism’s dynamically unfolding genesis. It is not read *into* the sensually perceptible phenomena, as Kant would have maintained (AA V:399), but rather read *from* them in a manner faithful to their phenomenal reality. When we (re)create the organism in the same way it creates itself, we recognize that the same idea lives both within us and within it.

In a summarizing statement from 1891, Steiner wrote:

“[I]t was Goethe’s great flight of thought that made him realize that one need not doubt the possibility of an explanation of the organic even if the inorganic laws of nature should prove inadequate for this purpose. Should our ability to explain only extend as far as we can apply the laws of the inorganic? What Goethe wanted was nothing other than to banish all dark and unclear ideas such as the life force, formative drive and so on from science and to find natural laws for them. But he wanted to find laws for organics in the same way as they had been found for mechanics, physics and chemistry, not simply adopt those existing in these other fields. (…) Goethe wanted an independent organic science that had its own axioms and its own method” (Steiner, 1989, GA 030:274-275).

And indeed, according to Steiner, Goethe’s morphological works “establish the theoretical foundations and methodology for studying organic nature” (CW 1:42; GA 001:70). Steiner maintained that Goethe “discovered *how one must think about the organism* in order to come to an understanding of it” (GA 028:112; italics added).

## Steiner’s Goethe-interpretation and intellectual intuition.

An organism can be characterized as a complex natural entity that dynamically forms itself according to holistic and purposeful laws. Accordingly, Kant identified two aspects as essential to the definition of an organism, distinguishing it from both man-made mechanisms and unorganized matter: purposive organization and self-forming force (*bildende Kraft*) (AA V:374). However, Kant’s theoretical discussion placed greater emphasis on purposiveness than on self-formation (cf. Frigo, 2009; Goy, 2012; Look, 2009).

Goethe in his “nature-based method” (*naturgemäße Methode*) simply followed the unfolding of the developing organism in which organizational laws and formative force are inseparably intertwined. Yet, with his characteristic precision, he also alluded – though only in passing – to law (that which nature “knows”) and force (nature’s “bringing forth”):

“[I]n describing the attempt at plant metamorphosis, a nature-based method had to be developed; for as the vegetation modelled its process before me step by step, I could not err, but had to acknowledge, by allowing it to proceed, the ways and means by which *it knows how to bring* the most enveloped state gradually to completion” (FA 24:442; emphasis added).

Steiner, in his analysis of Goethe’s approach, placed particular emphasis on the “*force* that calls itself into being” (CW 1:50; GA 001:83; emphasis added), that is, the productive entelechial principle of the organism. He maintained that in order to comprehend this principle, one must actively and imaginatively re-create the organism’s self-forming life, animate the idea (“bring it to life” within one’s mind [CW 1:3; GA 001:12]), and even endow it with a “substance” distinct from that which is perceived through the senses. In doing so, Steiner pointed to a mental productivity that gives rise to a content, which determines itself from within. As previously cited, Steiner referred to the apprehension of such self-determining ideal content as “intuition:”

“A concept that is not abstracted from the sensory world but whose content [*Gehalt*] develops out of itself and only out of itself can be called an *intuitive concept,* and the comprehension of such a concept may be called ‘intuitive knowledge.’ What follows from this is clear: *A living organism can be comprehended only through an intuitive concept*” (CW 1:50; GA 001:82-83).

Since Kant had denied the possibility of an intuitive grasp of an organism’s formative principle (AA V:407), it is precisely this capacity that is required in order to transcend the limitations he placed on the ability to know an organism. Achieving this, however, requires more than simply grasping the *laws* that govern an organism’s organization. One must directly perceive (intuit) the organism’s inner life, that is, the dynamic and productive *force* that drives its metamorphosis. Steiner maintains that this intuition becomes possible when the idea of the organism is “brought to life in one’s mind.” This *enlivening* of the idea is not merely cognitive but also creative, not merely a matter of seeing, but of doing.^29^

These issues bear resemblance to core themes in the philosophies of Goethe’s contemporaries Johann Gottlieb Fichte and Friedrich Wilhelm Joseph Schelling. Fichte claimed the conscious self-positing of the “I” as the foundation for all philosophy, while Nature to him was only “Not-I,” posited by the “I” as a limit of its own activity and a necessary condition for self-realization (Zöller, 1998). Schelling extended Fichte’s concept into a metaphysical idea of nature, which he understood as an expression of the “Absolute I,” the original unity of subject and object (Beiser, 2009). While for Fichte nature is reduced to a projection of the self and is never truly given in itself, in Schelling’s system the concrete “I” becomes a moment in the productive process of the Absolute, although the Absolute comes to self-consciousness in the human being.

Both Fichte and Schelling, however, claimed intellectual intuition as a methodological source of their systems, as extensively discussed by Förster in *The Twenty-Five Years of Philosophy*. In reference to Fichte, Förster explained that “the I knows its being as its deed, and this consciousness of the unity of thought and being is (…) a productive, an intellectual intuition” (Förster, 2012, p. 374). However, according to Förster, it is not possible to achieve an intellectual intuition *of nature*, as nature is not generated by the subject.^30^ He therefore argued that Goethe did not apprehend an organism through intellectual intuition, but rather through intuitive understanding (Förster, 2012, pp. 248–249). This implies that although intuitive understanding enables the cognition of the archetypal plant’s constructive element (the “leaf”) and constructive rule (expansion and contraction), it does not allow for a cognitive grasp of the constructor itself, that is of the self-generative, entelechial *force* by which the organism brings itself into being.

Steiner’s interpretation of Goethe extends beyond Förster’s account, for he asserted that in cognition of the entelechial principle of an organism “[o]ur mind must (…) generate not only the form, but also the content” (GA 002:109), a notion closely aligned with Fichte’s claim that in the self-positing of the “I,” activity and being are one and the same (Hoeltzel, 2020). In Steiner’s interpretation, it is the productive power of the cognizing subject that substantiates and enlivens the idea of the organism. Steiner asserted that it is within and through this mental activity that one gains conscious access to and participation in the generative, entelechial principle of the organism, that is in the “force that calls itself into being” (CW 1:50; GA 001:83). Steiner thus spoke of cognition of the organism just like Fichte spoke of cognition of the “I.”

This, however, is a significant notion. It asserts that we can consciously access the real and effective entelechial force of an organism by mentally re-creating its dynamic life-processes. That is why Steiner wrote:

“The idea of the organism, on the other hand, works actively within the organism as its entelechy – it is the essence of the entelechy itself in a form apprehended by our reason. The idea is not a summary of experience; it *brings about* that which is to be experienced” (CW 1:51; GA 001:84-85).

Steiner thus maintained that we cannot only grasp the holistic *laws* of an organism through intuitive understanding, but also experience the living, self-generating *force* of the organic through intellectual intuition. In this manner, not only the formative laws but the very force of the life of an organism become empirically accessible and thus open to legitimate scientific investigation (cf. Hueck, 2025).

## Conclusion

The question of how to explain living organisms remains a central concern in the philosophy of biology. Today, many researchers have moved beyond viewing organisms as genetically programmed, randomly selected survival-machines, and instead regard them as complex, purposively organized, self-generating, processual, autonomous, and agential wholes. Yet the challenge persists – not only to describe these characteristics, but also to provide a coherent explanation of them.

A fundamental prerequisite for explaining the self-forming capacity of organisms is its empirical observability. A natural efficacy which cannot, in some form, be observed or verified through observation cannot serve as the basis for scientific explanation. However, the life processes of an organism and their underlying force elude direct sensory perception. Through the physical senses, we can perceive only the outcomes – never the living, internally generative and purposively organizing force itself. For this reason, Kant regarded our concepts of life – such as purposiveness and self-generation – as of merely heuristic value, without ontological significance.

Kant’s perspective rests on the separation between perceptions (sensory intuitions) and concepts. In contrast, Goethe developed a method of observing and mentally re-creating living beings in which perception and concept are not held apart. For Goethe, “thinking was seeing, and seeing was thinking” (FA 24:595).

At the heart of Goethe’s method lies the conscious, active mental reconstruction of empirically observed organic processes of development and formation, accompanied by the observation and conceptual articulation of the insights gained through this engagement. Goethe’s approach may thus be characterized as phenomenological, imaginatively reproductive, and participatory.

Steiner elucidated this method in relation to – and in stark contrast with – Kant’s theory of knowledge, particularly as articulated in the *Critique of Teleological Judgment*. He emphasized the creative activity by which Goethe mentally re-enacted the living, formative processes of the organism, arguing that such active intellectual engagement gives rise to an intuitive apprehension of the organism’s entelechial principle. It is this emphasis on cognitive re-creation and the corresponding potential for intuitive insight into the generative forces of organic life that distinguishes Steiner’s interpretation of Goethe, setting him apart from other commentators. Through this reading, Steiner opened the way for recognizing and empirically observing spiritual (ideal) efficacy within physical (material) nature.

Finally, it is worth emphasizing that Goethe’s way of studying nature has inspired numerous researchers to apply it practically across various natural sciences (see references and link in footnote 4), and that Goethean science is increasingly being discussed within the context of an ecologically oriented and ethically grounded relationship to nature (Bauer, 2023; Brook, 1998, 2021b; Bywater, 2005; Nassar, 2022a; Suchantke, 2001).

## Footnotes

1 I use the following abbreviations: AA = Akademie Ausgabe of Kant’s collected works, volume and page number in the translation of J.C. Meredith (Kant, 2008); FA = Frankfurter Ausgabe of Goethe’s Schriften; WA = Weimarer Ausgabe of Goethe's Letters (Goethe, 2000); GA = Rudolf Steiner Gesamtausgabe (complete works; CW 1 is an Englisch translation by J.M. Barnes and M. Spiegler of GA 001 [Steiner, 2010]); SKA = Steiner Kritische Ausgabe. English readers will find many of Goethe’s texts from which I quoted in the excellent translation by Douglas Miller (Miller, 1995). However, translations of Goethe are my own and I did not also cite from Miller’s translation in order to avoid further increasing the number of sources.

2 In taking this position, I diverge from Eckart Förster, who in his seminal study *The Twenty-Five Years of Philosophy* (Förster, 2012) argued for a clear distinction between Fichte’s *intellectual intuition* and Goethe’s *intuitive understanding* (Förster, 2012, pp. 248–249). According to this distinction, Goethe attained an understanding of the principles and laws governing plant development, but not an intuition of the ontological reality of its self-generative force. See Sect. 7 for further discussion.

3 Explanation is a diverse topic in biology. A wide range of explanatory styles coexist (cf. Braillard & Malaterre, 2015 v.11; Craver, 2014; Potochnik, 2013; Ross, 2024), reflecting, in part, the diversity of questions posed and the differing assumptions about what constitutes a valid explanation (Morange, 2015). Here, I adopt an understanding of explanation in the Kantian sense as “derivation from a principle which must (…) be capable of being clearly cognized and specified” (AA V:412). For, as Kant asserted, “we have complete insight only into what we can make and accomplish according to our concepts” (AA V:384).

4 Goethe was not only a poet, but also an eminent natural scientist (Amrine, 2021; Amrine, et al., 1987; Hansen, 1907; Hassenstein, 1951; Heitler, 1970; Heusser, 2000; Magnus, 1906; Müller, 2015; Richards, 2010; Seamon & Zajonc, 1998; Semper, 1914; Steigerwald, 2002; Steigerwald, 2019; Zammito, 2017) and a philosopher of nature (Amrine, 2011; Bloch, 1952; Förster, 2012; Förster, 2014; Mehigan & Banki, 2020; Nassar, 2011; Nassar, 2022b; Tantillo, 2002; Weinhandl, 1932) with a strong emphasis on methodology (Bauer, 2023; Bortoft, 1996; Bortoft, 2012; Breidbach, 2006; Brook, 2021a; Califano, 2020; Förster, 2001; Goy, 2019; Heinemann, 1934; Hennigfeld, 2021; Hindrichs, 2011; Irwin & Baxter, 2008; Kuhn, 1982; Pfau, 2010; Robbins, 2006; Roy, 2018; Rupik, 2024; Sachtleben, 1994; Schieren, 1998; Simms, 2014; Stephenson, 1995; Vine, 2024; Wahl, 2005; Weik, 2017; Ziguras, 2014). There is a wealth of publications on Goethe’s science, especially on his morphology and theory of color (cf. the extensive bibliographies in Amrine, 1996; Amrine, et al., 1987; Danneberg, 2018; Schmid, 1940). Goethe’s morphological studies have been discussed by distinguished biologists, philosophers, and literary scholars (Arber, 1950; Cassirer, 1950; Heisenberg, 1968; Kuhn, 1988; Portmann, 1987; Russell, 1916; Simmel, 1906; Troll, 1925; Weinhandl, 1932; Weizsäcker, 1987; Wenzel, 1982, 1983; see also the collection in Engelhardt, 2024 of essays on Goethe from Carus, Cuvier, Du Bois-Reymond, Haeckel, Helmholtz, T.H. Huxley, Virchow, and others). Biologists who applied Goethe’s method have provided suggestions for understanding life, comparative morphology, development, and evolution (Bockemühl, 1985; Bockemühl & Suchantke, 1995; Bradley, 2011; Brady, 1984; Cornell, 1990; Ebach, 2005; Grohmann, 1959; Hoffmann, 2007; Holdrege, 2013; Holdrege, 2021; Hueck, 2023a; Kranich, 1999; Landman-Reiner, 2021a, 2021b; Riegner, 2013; Sassoon, 2018, 2020; Schad (ed.), 1982–1985; Schad, 2021; Stockmar, 1998; Suchantke, 2011; see also the extensive bibliography of Goethean science publications under https://science.goetheanum.org/forschung/eine-bibliographie).

5 Both books appeared at the Easter Book Fair in Leipzig, 1790 (Förster, 2012, p. 166; FA 24:937).

6 This developmental process unfolds according to an inner spatio-temporal logic: the first cycle (seed → shoot/leaves → calyx) is *successive*; the second (calyx → flower → reproductive organs) is *adjacent*; and the third (carpels → fruit → seeds) is *nested*. This intrinsic logic precludes a fourth cycle within a single plant, and thus, further development must begin anew from the seeds. – The interpretation of a plant’s metamorphosis as successive – adjacent – nested was first described by the botanist Friedrich Siegmund Voigt, who wrote about Goethe’s idea: “If, during the development of the plant, we observe the formation of parts one after the other, and in the flower we already observe a similar formation, more next to each other, then finally the fruit shows such a relationship in each other” (Voigt, 1817, pp. 440–441).

7 All translations of Goethe are my own.

8 In her detailed comparative analysis of various interpretations of Goethe’s concept of the “*Leaf*”, Ina Goy likewise concluded that the most compelling view is to understand it as a formal, changeable principle active in matter – one that is neither merely ideal (like a Platonic idea or a Kantian regulative concept) nor merely physical (like a gene-regulatory structure) (Goy, 2019). Goy justifies her decision by arguing that Goethe’s concept must be understood both as present in all parts of the plant and as subject to transformation. Gregory Rupik furthered this understanding by viewing the *Urpflanze* as the whole developmental “time-form” of a plant’s life-cycle, which cannot be summarized or distilled into a static schema, image, or Gestalt: “Like a melody, which can be grasped and appreciated as a whole only after its notes are played in sequence, the *Urpflanze* can be grasped after reading *The Metamorphosis of Plants*” (Rupik, 2024, p. 74).

9 The dual awareness of both the objects of study and the cognitive modes through which they are apprehended is characteristic of Goethe’s approach to natural science; cf., e.g., his *Writings on the Theory of Science* (Goethe, 1989, FA 25).

10 The botanist Wilhelm Troll remarked on this first discussion between Goethe and Schiller: “This conversation is of great symbolic importance, for in it Goethe’s world view touches on the system of the Königsberg philosopher” (Troll, 1925, p. 539; my translation). For discussion of Goethe’s notion of “seeing ideas with the mind’s eye” (cf. Förster, 2001; Hennigfeld, 2021; Hindrichs, 2011; Maatsch, 2012).

11 Wolff had already in 1769 observed the metamorphosis of plants, but in contrast to Goethe ascribed it to the physically obvious decrease in vitality [*Abnahme der Lebenskraft*] (Wolff, 1769 (lat.):62).

12 Jennifer Mensch wrote accordingly: Goethe’s “[i]ntuitive perception (…) cuts across Kant’s schema so far as it asserts the possibility of immediate sensible perception of the very idea preceding the parts as a whole” (Mensch, 2009, p. 15). However, as I will discuss in Sects. 6 and 7, Goethe’s intuitive perception is not sensible but intellectual.

13 For detailed comparisons of Kant’s and Goethe’s ways of knowing see Cassirer, 1944; Christensen & Brock, 2017; Gauss, 1969; Heinemann, 1934; Hindrichs, 2011; Maatsch, 2012; Nassar, 2015; Saman, 2023; Schieren, 1998; Seidel, 1948; Simmel, 1906; Troll, 1925; Vorländer, 1897, 1919; Weinhandl, 1932.

14 Goethe’s private collection harbored more than 18,000 specimens from geology, botany and zoology, together with many instruments from experiments in electricity and optics. Rudolf Magnus wrote about Goethe’s scientific work: “I can testify from personal experience to the extraordinary fascination of repeating Goethe’s experiments with his own instruments, of realizing the accuracy of his observations, the telling faithfulness with which he described everything he saw” (Magnus, 1906, p. 22). On the empirical abundance and accuracy of Goethe’s plant observations, see Hansen, 1907, and on his extensive geological studies Semper, 1914.

15 Goethe described several formative laws: polarity with progression (expansion, contraction, and metamorphosis); the double law [*doppeltes Gesetz*] of the inner constitution and outer modification of organisms (FA 24:357); the law of organic compensation (FA 24:836), according to which whatever is added to one part of an organism must be taken from another (a common idea of the eighteenth century, which even goes back to Aristotle [Cooper, 2022; Ziguras, 2010]). All these laws comprise a polarity, between which things unfold, exist, and progress, a concept which played a central role in Goethe’s worldview (Reill, 1986). Thus, Goethe claimed that “polarity and progression” [*Polarität und Steigerung*] are “the two great driving wheels of all nature” (FA 25:81).

16 For recent discussions of Goethe’s *Intuitive Power of Judgment* cf. Califano, 2020; Hennigfeld, 2021; Hindrichs, 2011; Holland, 2006; Maatsch, 2012; Mensch, 2009; Nassar, 2013; Ziguras, 2014.

17 For further discussion of Goethe’s “delicate empiricism” cf. Ebach, 2005; Robbins, 2006; Roy, 2018; Wahl, 2005.

18 For a detailed analysis of the mental faculties involved in the procedure of mentally re-creating a plant’s development cf. Hueck, 2025.

19 The image may be further enriched in a similar manner with additional properties, e.g., the plant’s physiology, proliferation, systematics, and evolution.

20 Steiner’s works are edited in the *Rudolf Steiner Gesamtausgabe* (complete works, GA). He gave more than 6200 lectures, approximately 4500 of which were transcribed, 3700 from co-stenographs (Hans Christian Zehnter, Rudolf Steiner Nachlassverwaltung, personal communication). Online translations of most of Steiner’s books and lectures can be found under https://rsarchive.org/. For a comprehensive list of Steiner’s publications on Goethe, see Steiner, 2022, SKA 1, p. 425 ff. For a growing collection of Steiner’s quotes on Goethe’s natural science, see https://publish.obsidian.md/steiner-goetheanism/INHALT. Except for quotes from CW 1, all other translations of Steiner’s texts cited here are my own.

21 Another fairly typical view dismisses Steiner’s commentaries on Goethe as merely aesthetic rather than scientific: “Rudolf Steiner, (…) without exploring Goethe’s systematic position, (…) argued in terms of an aesthetics of taste, but not in terms of a consistent theory of experience that truly absorbed the knowledge of the sciences” (Breidbach, 2008, pp. 100–101). – The following analysis of Steiner’s interpretation of Goethe’s morphological approach shows the superficiality of such scathing judgment.

22 Kant used the example of a ship moving on a river, in which its successive perceptions at different locations are connected by the concept of causality (AA III:B237). Cause (ship at position A) and effect (ship at position B) are both sensually perceptible, while the concept is a “mere means” of connection.

23 A similar consideration applies to the temporal relationships between the stages of plant development: “The living is a self-contained whole that sets its conditions from within itself. In the juxtaposition of the members, as in the temporal succession of the states of a living being, there is an interrelation that does not appear to be conditioned by the sensory properties of the members, not by the mechanical-causal determination of the later by the earlier, but which is governed by a higher principle that stands above the members and states. It is determined by the nature of the whole that a particular state is set as the first, another as the last; and the succession of the intermediate states is also determined in the idea of the whole; the before is dependent on the after and vice versa; in short, in the living organism there is *development* of the one from the other, a transition of the states into one another, no finished, completed being of the individual, but constant *becoming*” (CW 1:17–18; GA 001:34–35).

24 Steiner’s brief remark that the individual organs “exist for one another, but not as a result of one another” may be understood as an implicit correction of Kant’s dictum „that the parts of the [organic] thing combine of themselves into the unity of a whole by being reciprocally cause and effect of their form” (AA V:373). An organism’s parts are in fact *not* mutually cause and effect of their form; the form of the root is not the cause of the form of the leaves. The different organs condition each other, but they do not cause each other, because their cause lies above them in the non-sensory realm.

25 The concept of *intuition* plays a major role in Steiner’s philosophy and Anthroposophy (cf. Hueck, 2016).

26 A detailed account of such “bringing forth in intuition” of a plant’s development is provided in Sect. 5.

27 The critical observer may well ask how we can be certain that such a re-creation of natural phenomena is accurate and true. In other words: Can the results of Goethe’s scientific approach be falsified? The answer is: yes, of course. For Goethe’s method – like any other natural science – is grounded in precise empirical observation and must be tested against empirical evidence. Goethe himself described his method in his important essay *“Experience and Science”* as follows: “Once I have experienced the consistency and regularity of phenomena to a certain degree, I derive an empirical law from them and apply it to future occurrences. If the law and the occurrences subsequently fit perfectly, I have won; if they do not fit perfectly, I am made aware of the circumstances of the individual cases and forced to seek new conditions under which I can make the contradictory experiments more clear; but if, under the same circumstances, a case arises that contradicts my law, I see that I must press forward with all my work and seek a higher standpoint” (FA 25:125–126).

28 “An entelechy (…) is a force that calls itself into being” (CW 1:50; GA 001:83).

29 Reflecting on his early studies of Goethe, Steiner wrote in his autobiography: “The prevailing mindset of the age (…) seemed to me suited only to the formation of ideas about inanimate nature. I regarded its cognitive faculties as powerless to approach living nature. I told myself that in order to attain ideas capable of mediating cognition of the organic, it is necessary first to enliven the concepts of understanding, which are suited to inorganic nature. For they appeared to me lifeless – and thus fit only to grasp what is lifeless” (GA 028:112).

30 For a detailed discussion, cf. Nassar, 2013.
